# Prognostic Value of Microvessel Density in Head and Neck Squamous Cell Carcinoma: A Meta-Analysis

**DOI:** 10.1155/2020/8842795

**Published:** 2020-09-28

**Authors:** Yanbo Dong, Guangzhi Ma, Yukun Liu, Siyu Lu, Liangfa Liu

**Affiliations:** ^1^Department of Otolaryngology Head and Neck Surgery, Beijing Friendship Hospital, Capital Medical University, Beijing 100050, China; ^2^Department of Thoracic Surgery, West China Hospital, Sichuan University, Chengdu 610041, China; ^3^Department of Neurosurgery, Beijing Tiantan Hospital, Capital Medical University, Beijing 100070, China; ^4^Department of Emergency, Aviation General Hospital, Beijing 100012, China

## Abstract

The prognostic value of microvessel density (MVD) in head and neck squamous cell carcinoma (HNSCC) remains disputable. The purpose of this study was to comprehensively determine the prognostic value of MVD in HNSCC. Relevant literatures were identified using PubMed, Embase, and Cochrane Library. A meta-analysis was performed to clarify the prognostic role of MVD in HNSCC patients and different subgroups. A total of 14 eligible articles were included in this meta-analysis. The combined hazard ratio (HR) and 95% confidence interval (95% CI) for overall survival (OS) of 11 studies was 1.663 (1.236-2.237, *P* = 0.001), and the pooled HR and 95% CI for progression-free survival (PFS) of 7 studies was 2.069 (1.281-3.343, *P* = 0.003). Subgroup analyses were also performed on different issues, such as regional distribution of patients, age, tumor location, antibody, and treatment strategy. To conclude, high MVD is associated with worse OS and PFS in patients with HNSCC.

## 1. Introduction

As the sixth leading malignancy worldwide, head and neck squamous cell carcinoma (HNSCC) had an annual incidence of more than 600,000 cases [1]. It encompasses cancers of oral cavity, pharynx and larynx, and upper aerodigestive tract [2]. The disease distinguished itself from others by the following characters: complicate anatomy of the primary tumor sites, regional or distant metastasis, the concurrency of secondary primary tumors, local tumor recurrence, and detection at late stages. Despite considerable advancements in diagnostic and treatment practices that have been made, the 5-year overall survival rate of HNSCC still remains unsatisfactory [3]. Globally, HNSCC incidence trends vary greatly by geographical areas, reflecting differential trends in tobacco usage, alcohol abuse, and sexual norms across countries. For instance, Eastern Europe has the world's highest cigarette consumption rates among both men and women [4]; thus, HNSCC incidence is on the rise, and mortality is the lowest in Europe [5]. In China, smoking prevalence among men is greater than 50%, whereas prevalence among women in the country is at least 10 times lower than among men [6]. In the USA, the epidemiology of HNSCC changes dramatically because of the increasing incidence of HPV related HNSCC, and declining tobacco-abusing prevalence rates paralleled by a decrease of HPV-negative HNSCC [7]. 5-year survival of HNSCC patients in the USA was approximately 65.0%, significantly increasing for all primary sites over the past 20 years from 54.7% in 1992 to 1996, to 65.9% in 2002 to 2006 [8]. The increase was attributed to advances in treatment, and HPV-positive HNSCC had a favorable prognosis.

Apart from well-known prognostic factors such as tumor size, histological invasion depth, lymph node metastases, and tobacco abuse (smoking or chewing), there were specific prognostic factors for HNSC from different primary sites, such as alcohol consumption for oro-hypopharyngeal carcinoma [9], human papillomavirus (HPV) infection for oropharyngeal carcinoma [10], and plasma Epstein-Barr virus (EBV) DNA for nasopharyngeal carcinoma [11]. Besides, many prognostic markers have been identified to affect the outcomes of the disease as well, such as p53, Ki-67, p16, Cyclin D1, and microvessel density (MVD) [12, 13]. MVD proved a reliable marker for identifying recurrence in cancer patients and was regarded as an alternate marker for tumoral angiogenesis more than 10 years ago [14]. The correlation between the incidence of metastasis and tumor angiogenesis as measured by MVD was first described in patients with breast cancer by Weidner et al. [12]. The study generated much interest in MVD among oncologists and initiated a search for similar associations in a diversity of other neoplasms. Subsequently, increasing evidence showed prognostic value of MVD in patients with nonsmall-cell lung cancer [15], colorectal cancer [16], head and neck cancer [17], etc.

Currently, routine antibodies used for angiogenesis staining in solid tumor include those against platelet/endothelial cell adhesion molecule CD31, pan-endothelial marker CD34, homodimer trans-membrane protein CD105 (Endoglin), and von Willebrand Factor (factor VIII) [18]. To assess MVD quantificationally, Weidner et al. also put forward a method called “hot spot” to identify the area of highest vascular density (hot spot) by light microscopy and then count individual microvessels at a higher power (×200 magnification field) [12].

The prognostic value of MVD in HNSCC was reported in multiple studies, and many suggested MVD a crucial predictive factor in tumor progression and metastasis [17, 19], whereas some researchers did not reach to any conclusive result indicating MVD's prognostic value for HNSCC [20, 21]. Due to those inconsistent results above, we aimed to perform a systematic review and meta-analysis of all available literature relating MVD to comprehensively determine the prognostic value of MVD among HNSCC patients.

## 2. Materials and Methods

### 2.1. Literature Searching

This study being a meta-analysis, institutional review board approval is waived for this study type at our institution. Two reviewers (YD and GM) independently searched all studies targeting all angiogenesis markers of HNSCC, by an online search using PubMed, EMBASE, and Cochrane Library. A search strategy combining the terms (otolaryngology or “head and neck” or buccal or mouth or “oral cavity” or lip or tongue or larynx or hypopharynx or oropharynx or nasopharynx) and (cancer or “squamous cell carcinoma” or neoplasm or tumor or carcinoma or “squamous carcinoma”) and (MVD or “micro vessel density” or “microvessel” or angiogenesis or CD34 or CD31 or CD105 or ENG or UEA or CD55 or “factor VIII” or f8) and (prognosis or survival or mortality) was developed. The last query was updated on February 29, 2020.

### 2.2. Inclusion Criteria

Eligible studies should meet all the criteria as follows. In studies on head and neck cancer, all included patients should be confirmed with squamous cell carcinoma; MVD was estimated and its association with prognosis was reported. Data provided within the literatures were feasible for log hazard ratio (logHR) calculation, according to methods by Parmar et al. [22] and Williamson et al. [23]. Eligible study categories include cohort studies and case-control studies.

### 2.3. Exclusion Criteria

Literatures should be excluded if any of the following was matched: case reports, conference abstracts, studies on animals, in vitro studies, or any other types of laboratory studies, review or systematic review, and studies failing to clearly report the data that met our interest.

### 2.4. Data Extraction

Two reviewers (YD and YL) extracted data from all original studies independently. The primary data were hazard ratio (HR) and 95% confidence interval (95% CI) of HR. Additional data obtained from the studies included names of the first author, publication year, country, median or mean age of patients involved, number of patients, gender, primary site of cancer, clinical stage, antibodies applied for immunohistochemical staining, treatment strategies, and evaluation of high MVD.

The primary data for calculation were multivariate/univariate Cox hazard regression analysis or HR with 95% CI for overall survival (OS), progression-free survival (PFS), or disease-free survival (DFS). OS was defined as the time from diagnosis of HNSCC until death from any cause. PFS was defined as the time from diagnosis of HNSCC until progression of the disease or death from any cause. DFS was defined as the time from complete removal of the tumor to recurrence or progression. The literature selection and data extraction were performed by 2 reviewers (YD and YL) independently, with any discrepancies being discussed and reassessed.

### 2.5. Methodological Assessment

Quality of each study was assessed according to the Newcastle-Ottawa Scale (NOS) criteria [24]. Three aspects of each study were evaluated as follows: subject selection: 0 to 4, comparability of subject: 0 to 2, and clinical outcome: 0 to 3. The total score ranged from 0 to 9; a study that scored 6 or more was eligible for data-pooling and any literature that scored 7 or more was considered of good quality. The whole evaluation process was conducted by 2 reviewers (YD and SL) independently.

### 2.6. Statistical Analysis

The software STATA (version 12.0; Stata Corporation, College Station, TX) was applied for data analysis. LogHR and its standard error were extracted for pooling the survival results, but they were not provided directly in most articles. In that cases, we could apply the HR and its 95% CI for calculation of LogHR and the standard error, according to methods by Parmar et al. [14] and Williamson et al. [15]. The HR and 95% CI reflected the overall prognostic value of MVD. An HR >1 indicated poor survival of HNSCC patients with relatively high MVD and would be considered statistically significant if the 95% CI did not overlap 1. HR values of MVD from multivariate survival analyses were prior used if HR values of both univariate and multivariate analyses were provided. Adjusted HR was first applied if adjusted and unadjusted HRs all existed. The heterogeneity assumption of pooled HRs was assessed by *I*^2^ index and *P* value [25]. The fixed-effect model (the Mantel-Haenszel method) [26] was applied if the heterogeneity between studies was not statistically significant (*P* > 0.10 or *I*^2^ < 50%). If else, to reduce the impact of heterogeneity, HR should be evaluated by the random-effect model. Forrest plots were used to estimate the effect of high MVD counts on survival outcome. Publication bias was assessed using the Begg funnel plot and the Egger test [27]. For Egger's test, *P* < 0.1 was considered to be statistically significant. Trim and fill analysis was applied if there was publication bias. For other analyses, *P* < 0.05 was considered to be statistically significant.

## 3. Results

### 3.1. Study Selection

**Figure 1:**
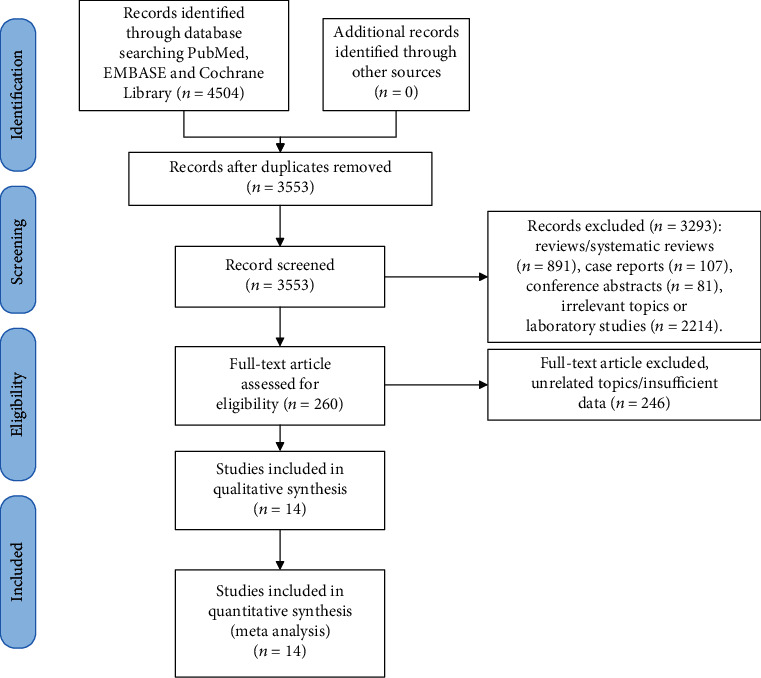
Flow diagram of literature search.

A total of 3,553 studies (after duplicates removal) were retrieved by our initial literature search. Abstracts of each literature were carefully read and screened. Exclusion reasons and numbers of studies were as follows: irrelevant topics or laboratory studies (*n* = 2214), reviews or systematic reviews (*n* = 891), case reports (*n* = 107), and conference abstracts (*n* = 81). Totally, 260 potentially eligible studies were obtained and scrutinized; 246 of them were omitted because of the following reasons: 87 studies without HR estimation of MVD, 78 studies without follow-up time, 46 studies whose data were either survival curve or illegible of HR estimation, 33 studies without full article, and 2 studies with scores of NOS lower than 6. Finally, 14 observational (cohort and case-control) studies (*n* = 1,638 patients) met our inclusion criteria and were capable of data extraction as well as meta-analysis. The flow diagram of literature selection was presented in Figure 1.

### 3.2. Study Characteristics

Among the 14 eligible studies, 5 were from Asia, including 3 from China and 2 from Japan; 6 were from Europe, including Italy, Finland, Greece, Switzerland, and Turkey. North American patients that were from the USA comprised the rest population of included studies. Altogether, 1,638 patients were included, with a majority of male patients. All cases included were HNSCC with various primary sites such as oral cavity, pharynx and larynx, and tumor stages varied from I to IV. Antibodies applied for immunohistochemical staining were against CD105, CD31, CD34, or Factor VIII. All studies remarked on HRs that were feasible for data-pooling. High MVDs were assessed quantitatively or defined through intensity levels of staining. All eligible studies scored no less than 6. To conclude, baseline information was summarized in Table 1.

### 3.3. Meta-Analysis Results

**Figure 2:**
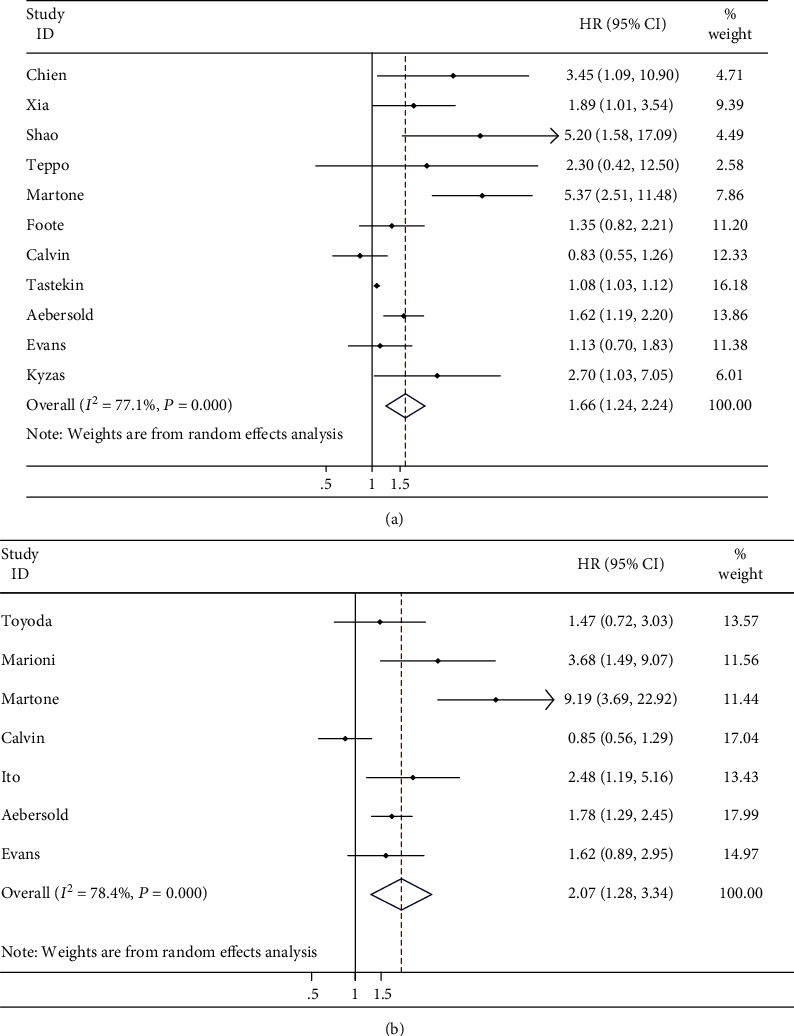
Meta-analysis. (a) Estimated hazard ratio (HR) summary for overall survival (OS) in all patients. (b) Estimated HR summary for progression-free survival (PFS) in all patients.

The prognostic value of MVD was valued by OS and PFS. The correlation between them was determined according to combined HRs and related 95% confidence intervals. Consequently, the prognostic value of high MVD for OS was analyzed in 11 studies, with the combined HR of 1.663 (95% CI: 1.236-2.237, *P* = 0.001), and the combined HR for PFS was 2.069 (95% CI: 1.281-3.343, *P* = 0.003), suggesting a negative impact of MVD on HNSCC prognosis. The scores of *I*^2^ of heterogeneity test were 77.1% and 78.4%, respectively; accordingly, random-effect model was adopted (Figure 2).

### 3.4. Subgroup Analysis

In accordance with basic information and extracted data from all eligible literatures, subgroups were sorted due to regional distribution of patients (Asia, Europe, and North American), median age (≥60 years), tumor location (oral cavity and pharynx), antibodies for staining (CD105), and treatment strategy (surgery and chemoradiotherapy).

#### 3.4.1. Regional Distribution of Patients

Altogether, among the 11 studies on MVD and OS, 5 were from Europe, 3 were from North America, and 3 were from Asia. The combined HR for OS in Europe was 1.979 (95% CI: 1.174–3.334, *P* = 0.010), with a significant heterogeneity (*I*^2^ = 85.7%, *P* < 0.001), and random-effect model was applied. The combined HR for OS in North American was 1.049 (95% CI: 0.805–1.367, *P* = 0.725), heterogeneity was insignificant (*I*^2^ = 13.9%, *P* = 0.313), and fixed-effect model was applied. With regard to Asian patients, heterogeneity was not found and the pooled HR for OS was 2.530 (95% CI: 1.534–4.174, *P* < 0.001, *I*^2^ = 20.6%).

#### 3.4.2. Age

Median or mean age was provided in 10 studies that were all over 60 years old. Half of them provided the relation between MVD and OS, while the other half on MVD and PFS. Combined HR for OS and PFS was 2.238 (95% CI: 1.213-4.130, *P* = 0.010) and 2.728 (95% CI: 1.492-4.986, *P* = 0.001), respectively. Heterogeneity was significant, and random-effect model was applied.

#### 3.4.3. Tumor Location

Primary sites of HNSCC in the given literatures contained oral cavity, nasopharynx, oropharynx, hypopharynx, larynx, and a combination of them. The pooled HR of pharyngeal cancer for OS was 1.390 (95% CI: 0.993-1.945, *P* = 0.055) including 4 studies. In addition, HR of oral cancer for OS was 2.748 (95% CI: 1.053-7.170, *P* = 0.039) including 2 studies. Heterogeneity was significant and random-effect model was applied.

#### 3.4.4. Antibodies for Immunohistochemical Staining

Antibodies against CD105 were used within 4 studies for vasculature staining. The combined HR was 2.916 (95% CI: 1.945, 4.370, *P* < 0.001). Heterogeneity was not detected, and fixed-effect model was used (*P* = 0.219, *I*^2^ = 32.2%).

#### 3.4.5. Treatment Strategy

Treatment strategy of HNSCC in these eligible studies included surgery and chemoradiotherapy. Patients in 7 of the studies received surgical treatment, while patients in 4 of them received chemoradiotherapy. The pooled result for OS was also indicative, HR for the former was 2.578 (95% CI: 1.522-4.365, *P* < 0.001), and heterogeneity was significant statistically (*P* = 0.016, *I*^2^ = 61.5%). For the latter, the HR was 1.176 (95% CI: 0.917-1.508, *P* = 0.202), and heterogeneity was statistically significant (*P* = 0.027, *I*^2^ = 67.2%). Random-effect model was used because of significant heterogeneity.

All summarized results were presented in Table 2.

### 3.5. Sensitivity Analysis and Publication Bias

**Figure 3:**
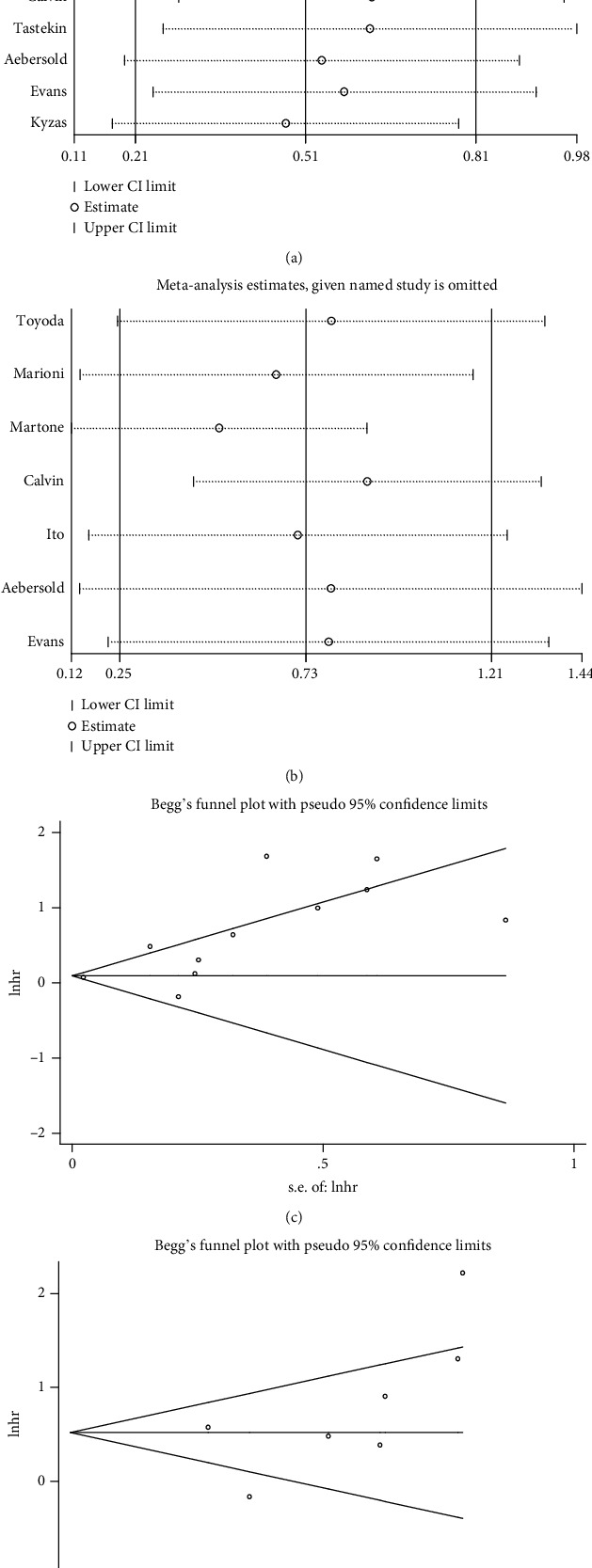
Sensitivity analyses and Begg's publication plots. (a, b) Sensitivity analysis results on omission of each individual study for corresponding meta-analysis in Figures 2(a) and 2(b). (c, d) Funnel plots of publication bias summary for corresponding meta-analysis in Figures 2(a) and 2(b).

**Figure 4:**
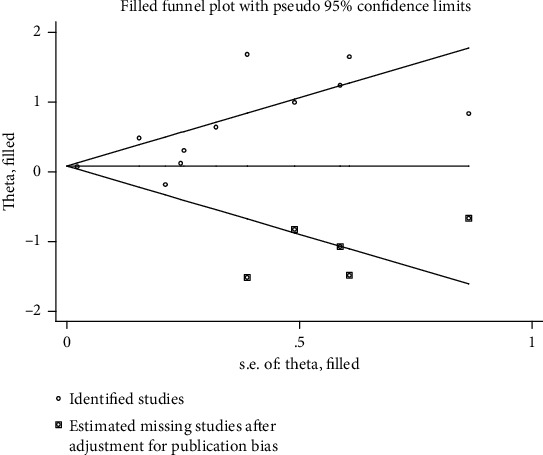
Trim and fill analysis for OS in all patients.

As the sensitivity analysis showed, the combined results representing the pooled HRs for OS and PFS did not change prominently when each study was removed sequentially (Figures 3(a) and 3(b)). This demonstrated the above-pooled results convincing and steady. However, publication bias was found for OS by visual inspection of Begg's funnel plot (Figure 3(c)) and the Egger's test (*P* = 0.003). While Begg's funnel plot (Figure 3(d)) for PFS did not show obvious publication bias with Egger's test *P* = 0.168. We then applied Trim and fill method to correct the result of pooled HR for OS (Figure 4). Five potentially missing studies were replaced and the adjusted HR was 1.086 (95% CI: 1.041-1.132).

## 4. Discussion

This study aims to identify the prognostic value of MVD among HNSCC patients by data pooling and meta-analysis. Resultantly, the pooled HR (95% CI) for OS was 1.663 (1.236-2.237), and the pooled HR (95% CI) for PFS was 2.069 (1.281-3.343). High MVD proved an adverse prognostic factor that shorten OS.

In consideration of regional distribution of patients, subgroup results demonstrated that MVD was a negative prognostic marker for European and Asian patients. Conversely, MVD was not related to outcomes about North American participants. The reason for the difference of MVD's prognostic value by geographical areas was possibly related to the epidemiology of HNSCC. In the USA, tobacco-using prevalence rates as well as HPV-negative HNSCC is declining, while the incidence of HPV positive HNSCC is rising. And, HPV-positive HNSCC tends to have a favorable prognosis. Therefore, the prognostic role of MVD might be influenced by the HPV status. However, no direct evidence could support this primitive deduction so far [28].

When it comes to age subgroup of which studies with patients' median/mean age ≥60, MVD was in correlation with poor OS and PFS. As for HNSCC patients whose primary site of tumor was oral cavity or intratumoral vessels stained by CD105, high MVD was also a poor prognostic factor. Similar results were also found in patients receiving surgery as major treatment strategy. On the contrary, for several studies involving patients with pharyngeal cancer or those treated with chemoradiotherapy, integrated data through multivariate analyses manifested that MVD was not an adverse prognostic marker statistically for HNSCC.

Remodeling of the vessels and formation of new ones is one of the hallmarks of tumorigenesis [29]. Tumor angiogenesis is a complicated biological process participated by numerous angiogenetic factors in tumor microenvironment [30]. VEGF was deemed to be the most important factor [31]. Previous studies tried to identify the correlation between VEGF and MVD in HNSCC and to identify the prognostic value of both. However, no agreement was reached. A few studies reported a positive relation between VEGF expression and MVD [32]. On the contrary, many studies showed VEGF level correlated with MVD negatively [33, 34]. Similarly, the prognostic value of both VEGF was debatable [13]. Therefore, more studies are required to provide evidence on the question and MVD values should be referred along with VEGF expression level to estimate the angiogenesis status of HNSCC cases.

MVD reflects the intensity of angiogenesis within the tumor and can be identified through routine pathology methods by IHC. A high degree of feasibility and availability makes it a practical way of clinical use for the evaluation of neoplastic vascularization. However, several issues should be considered in regard to MVD. There are some restrictions of the parameter itself. Firstly, identification and evaluation of MVD was mainly based on subjective assessment, such as “hot-spot” method and vessel-counting [35]. It is difficult to avoid subjective bias from pathologists, especially in different studies. Secondly, MVD value was generated from tissue sections; thus, it could not reflect the tumor status thoroughly. Last but not least, no consensus had been reached on the best antibody for MVD IHC staining. Although the pan-endothelial markers, such as CD31 and CD34, were generally applied to evaluate tumor vascularity, they did not differentiate newly formed and preexisting vasculature [36–38]. However, CD105 appeared exclusively correlated with the endothelial cells in the newly formed vessels and the immature tumor vessels [39, 40]. Interestingly, CD105 was used in 4 of our screened studies, and subgroup analysis showed a positive result between MVD stained by CD105 and OS, with insignificant heterogeneity. Despite the disadvantages mentioned above, MVD was still a widely used method to assess angiogenesis quantitatively [12].

The publication bias was one of the major concerns for all meta-analysis. Begg's funnel plot and Egger's test indicated that significant publication bias was found in pooled HR for OS group. But no publication bias was found in PFS group or almost all subgroup meta-analyses other than the European subgroup (Egger's test *P* = 0.047). Trim and fill method was used to correct the result. Positive results were acquired after correction, suggesting them robust and reliable notwithstanding potential publication bias. We tried to decrease publication bias by making a complete literature search. However, we have to exclude the few studies published in languages other than English. Another source of publication bias may be that nonsignificant results might not be reported [41–43].

There was a meta-analysis on prognostic value of MVD in HNSCC published in 2014 [21]. Yu et al. integrated 13 studies and investigated the risk ratio of high MVD on 5-year OS. However, they did not find MVD a biomarker for OS of HNSCC with a pooled risk ratio of 1.23 (95% CI: 0.99-1.52, *P* = 0.06). According to their analysis, MVD with lymph node (LMVD) was associated with worse 5-year overall survival (OS) (RR, 2.07; 95% CI: 1.16-3.71). This result was also meaningful and provocative, because the occurrence of lymph node metastasis in HNSCC is prevalent and of independent poor prognostic factor. But they pooled only 5 studies (*n* = 408 patients) for LMVD, which lacked adequate evidence and was less persuasive. What is more, this article estimated survival data from publications which did not directly provide HR and 95% CI. To limit the potential risk of bias, those literatures without HR and 95% CI were omitted in our study. In addition, our systematic review and meta-analysis involved 14 publications incorporating 1,638 HNSCC patients, which had the largest data so far in scale.

We acknowledge that some limitations exist in our study. Firstly, heterogeneity of the results. Sensitivity analysis and subgroup analysis were conducted in terms of several aspects, but the source of heterogeneity was still vague. Therefore, the heterogeneity might come from the inconsistency of baseline characteristics from included studies such as patients' regional distribution, tumor stage, treatment strategies, and immunohistochemistry (IHC) details including different cut-off values and antibody utilization. Tumor stage might correlate with MVD level, but lack of original data made it impossible to determine its prognostic value in every single stage. Secondly, the number of included literatures in our study is not big, especially in the analysis of PFS. If more evidence were available, the current study could be reconducted. What is more, only English databases were searched. Notwithstanding the above shortcomings, bias was controlled to the minimum with the effort of prudently pooled statistics and detailed protocols, and the results of this study were ensured credible.

## 5. Conclusions

This meta-analysis identified that high MVD was potentially correlated with worse 5-year OS and PFS in HNSCC patients. It is inconsistent to define MVD levels in current IHC criteria, which might be one of the sources of heterogeneity. More fundamental studies and randomized controlled trials with larger samples are required to validate the prognostic role of MVD for HNSCC patients.

## Figures and Tables

**Table 1:** Characteristics of the eligible studies of MVD and HNSCC.

Author	Year	Country	Median/mean age	*N* (F/M)	Clinical stage	Location	Antibody	Evaluation of high MVD	Quality score
Chien [44]	2005	China	55.2	73 (1/72)	II-IV	HP	CD105	11.94/MPF	8
Toyoda [45]	2015	Japan	68	70 (9/61)	III/IV	HP	CD34	13/HPF	7
Xia [46]	2014	China	60	87 (38/49)	I-IV	OC	CD105	19/MPF	8
Shao [47]	2008	China	—	59 (24/35)	I-IV	OC	CD34	80/MPF	6
Marioni [48]	2011	Italy	72.2	57 (5/52)	I-IV	L	CD105	IL	7
Teppo [49]	2003	Finland	67	100 (15/85)	I-IV	L	CD31	9.6/HPF	8
Martone [50]	2005	Italy	60.8	127 (5/122)	I-IV	OC, OP, L	CD105	20.2/MPF	8
Foote [51]	2004	USA	—	123 (34/89)	I-IV	NP	F8	60/MPF	7
Calvin [52]	2007	USA	—	450 (96/354)	III/IV	OC, OP, HP, L	F8	60/MPF	7
Ito [53]	2001	Japan	61	43 (13/30)	III/IV	OC, OP, HP, L	CD31	30/MPF	6
Tastekin [54]	2015	Turkey	59.48	46 (10/36)	I-IV	OP	CD34	IL	7
Aebersold [55]	2002	Switzerland	57	95 (23/72)	I-IV	OP	CD31	IL	8
Evans [56]	2018	USA	61	200 (70/130)	I-IV	OC, OP, L	CD31	PFS:53/HPF; OS:39.667/HPF	6
Kyzas [57]	2006	Greece	64.5	108 (20/88)	I-IV	OC, L	CD105	49/MPF	6

F: female; M: male; OC: oral cavity; HP: hypopharynx; OP: oropharynx; NP: nasopharynx; L: larynx; F8: factor VIII; IL: intensity level; MPF: ×200 magnification field; HPF: ×400 magnification field.

**Table 2:** Meta-analyses of MVD and survival of HNSCC.

	N of studies	Model	HR (95% CI)	Log rank *P*	Heterogeneity (*I*^2^, *P*)
Total OS	11	Random	1.663 (1.236, 2.237)	0.001	77.1%, <0.001
Total PFS	7	Random	2.069 (1.281, 3.343)	0.003	78.4%, <0.001
European OS	5	Random	1.979 (1.174, 3.334)	0.010	85.7%, <0.001
North American OS	3	Fixed	1.049 (0.805, 1.367)	0.725	13.9%, 0.313
Asian OS	3	Fixed	2.530 (1.534, 4.174)	<0.001	20.6%, 0.284
Median/mean age ≥ 60 OS	5	Random	2.238 (1.213, 4.130)	0.010	67.3%, 0.016
Median/mean age ≥60 PFS	5	Random	2.728 (1.492, 4.986)	0.001	68.2%, 0.014
Pharynx OS	4	Random	1.390 (0.993, 1.945)	0.055	73.8%, 0.010
Oral cavity OS	2	Random	2.748 (1.053, 7.170)	0.039	54.1%, 0.140
CD105 OS	4	Fixed	2.916 (1.945, 4.370)	<0.001	32.2%, 0.219
Surgery OS	7	Random	2.578 (1.522, 4.365)	<0.001	61.5%, 0.016
Chemo-radiotherapy OS	4	Random	1.176 (0.917, 1.508)	0.202	67.2%, 0.027

HR: hazard ratio; CI: confidence interval; OS: overall survival; PFS: progression-free survival; N: number.

## Data Availability

All data generated or analyzed during this study are included in this published article.
